# Cases in Practice

**Published:** 1858-10

**Authors:** Thos. W. Fry

**Affiliations:** Crawfordsville, Ind.


ARTICLE III.

### CASES IN PRACTICE.

BY THOS. W. FRY, M. D., CRAWFORDSVILLE, IND.

CASE 1.—In the year 1842, I was called to see Miss S., and found her in violent universal convulsions. She was at that time about eighteen years of age, and had been subject to convulsions from her fourteenth year. When first attacked she was living in Lowell, Massachusetts, and had been attended by the physicians of that city and the city of Boston. Her first attacks were so violent, and left her in a state of such stupor, as to induce the belief in her attending physicians that the convulsions resulted from some disease of the brain, and were, therefore, incurable. Her mind remained unimpaired, excepting the constant dread, which was a perpetual cause of depression. The reception of unexpected news, whether joyous or the reverse, any startling noise, a sudden flushing of the cheek, would invariably excite fears of an attack.

A careful examination of the case after the convulsion had passed off, and frequently during the intervals, forced the conviction upon me that the brain was not primarily affected, but merely responded to reflex action of other more diseased organs. To my surprise, I found that the menstrual functions were normal and regular, accompanied with no unpleasant or alarming symptoms. The bowels were very irregular in their action, generally bound, and the appetite variable. The spinal column was exceedingly tender and gave great pain on pressure, more especially through the regions of the heart, stomach and lungs. Spinal irritation I therefore regarded as the true seat of the disease, and the prime cause of the convulsions.

The impressions made upon her mind by her former physicians that the disease had its seat in the brain, and was, of consequence, incurable, produced a despondency from which it was difficult to arouse her. She looked forward to a life of physical suffering and mental imbecility, which threw a shade of darkness over her entire being.

The treatment of this case was as much mental as physical. By the strongest assurances that her brain was not primarily diseased, and that spinal irritation might be greatly benefited if not entirely relieved, I succeeded, in some degree, in dispelling the depression under which she had been so long laboring, and inspiring a faint hope of ultimate recovery.

Her bowels were regulated by the use of an occasional alterative, and a simple pill, composed of equal parts of rhubarb, aloes and castile soap. But the spinal irritation was more difficult to control. Cupping on either side of the spine, irritating ointment and plasters, hot soap suds and liniments were alternately used, which gave gradual but almost perfect relief. The intervals between the convulsions became longer until they entirely ceased. She has been married for some ten or twelve years, has had several severe attacks of illness, but no return of convulsions. They became less frequent and less severe as the spinal irritation subsided.

The importance of this case consists not so much in the character of the treatment or the remedial agents employed, as in showing the necessity of a careful and correct diagnosis before pronouncing so certainly on the incurability of disease in the vital and more important organs.

CASE 2.—Mr. C. W., a very promising young man, having completed his collegiate course in Wabash College, and desiring to prepare himself for the ministry, left his home in the West in 1848, and entered upon his studies with much devotion and energy in Harvard University. He had not been many weeks in the school before the cold of winter set in with great severity. The keen, piercing winds proving too severe for his lungs, a hemorrhage occurred, which prostrated his strength and greatly excited the fears of himself and his friends. Several attacks came on in quick succession, followed by such extreme prostration as to render the farther prosecution of his studies absolutely impossible. Having a physician friend and relative near Boston, he repaired to his home to be treated conjointly by his friend and Dr. Jackson, of Boston. The enlarged experience of Prof. Jackson, his deservedly high reputation in detecting diseased lungs, by percussion and auscultation, gave importance and weight to his opinions. After a careful examination, the Doctor gave it as his opinion that the patient would, in all probability, die within three months and could not possibly live two years; that his lungs were diseased beyond the hope of being reached by remedial agents. This opinion, pronounced with such decision, by one who was regarded as almost infallible in such cases, rested like a mountain weight on the spirits of the young man, and after resting for a short time and gaining a little strength, came by gentle stages to his father's home, without hope and with no other prospect than that of an early death. It was with much difficulty that either he or his friends would consent to the use of any remedial agents, thinking that they would only tend to hasten the last dreaded event. But the remedies which his physicians had used were instrumental in arresting the hemorrhage, and he yielded to the argument that the remedy which would arrest might prevent. I again and again examined the case with all the care and all the lights in my possession, and as often expressed the belief that his life might and doubtless would be spared; that a residence in the southern part of our country would, in all probability, exert such healing influence on his lungs as to enable him to reach the alloted age of man. The ordinary anodyne and astringent remedies were resorted to, in connection with a wholesome, moderately nutritious diet; and with but an occasional slight attack of hemorrhage, he passed the much dreaded period of three months, apparently in a much better condition than when he first reached home. Living beyond this time inspired him with a little stronger hope, that a southern residence and the use of similar remedies might carry him beyond the two years, through which it was thought impossible for him to live. He remained at his father's during the winter, spring, summer and part of the fall months, with an occasional hemorrhage, but with evident improvement in both body and spirits. When the cold weather of fall came on he left for the South, went directly to New Orleans, thence by the Gulf to Mobile and up the Alabama River, for the purpose of spending the winter with an old friend and physician, who resided in a village a little north of Mobile. Dr. —— examined his chest, corroborated my opinion, and gave even stronger encouragement than I had done; he was a man of long experience, great intelligence and superior skill, which served to excite still brighter hope in the patient's breast.

The attacks of hemorrhage occasionally came on even in that mild climate, and were followed with a degree of prostration which induced him to give up all hope of the ministry. He now turned his attention to the study of medicine, his health gradually improved, his strength increased, his attacks became less severe and less frequent; at the end of twelve months he was enabled to attend a partial course of lectures in New Orleans. The improvement continued until he completed a thorough course of medical instruction, received the honors of the New Orleans school, settled in that city, is rapidly rising not only in the estimation of the people but of his professional brethren, and bids fair for a long life of prosperity, usefulness and honor.

He has passed through several epidemics of yellow fever, has been brought to the verge of the grave by that terrible scourge, but still lives, an ornament and an honor to our profession. It should be stated that he made a very free use of cod liver oil, and became so fond of it as to use it on his salad in place of olive oil.

The history of this case is given for the purpose of showing the necessity of continuing our efforts for the relief of cases almost hopeless, and even when opinions against the possibility of recovery have been given by the most experienced and skilful. It was more difficult to combat the mental depression produced by Prof. Jackson's opinion than it was to meet and alleviate the symptoms of physical disease.

CASE 3.—In 1855 I was called to visit Mrs. B., who in early life had a fine, robust constitution, enjoyed almost perfect health, and her spirits were bright and buoyant. At the time, she had been married some ten years and had given birth to seven children, most of whom were still-born or died in early infancy. The physical suffering and mental anxiety through which she had passed had greatly impaired her constitution, destroyed her health and crushed her spirits. For some year or two previous to her removal to Crawfordsville she had been treated upon what is commonly called the heroic plan, which proved illy suited to her case. Bottle after bottle of strong, nauseating medicines were administered, which served only to diminish the strength, reduce the system and deprive the patient of all relish for food. While *enciente* with her last child, the physician pronounced her incapable of bearing it to the full period of gestation, and with the view of saving the mother's life, endeavored to produce abortion by rupturing the membranes. But instead of passing the instrument into the os uteri and then through the membranes, he plunged it into the walls of the uterus; hemorrhage, of course, followed, but the fœtus remained untouched and unharmed, and grew on to its full period, was born in full health and vigor, and is now a boy near three years old and is particularly bright. After the delivery, the mother was treated in the same severe manner, and was reduced so low that she gave up all hope of recovery; believing herself the subject of an incurable disease, fearing that she would soon become a burden to her family and friends, hopeless and despairing, she longed for death to release her from her present sufferings. The very thought of medicine had become disgusting to her, and the word "*doctor*" was repulsive to her every feeling.

Having removed from the city to her present home, she fondly hoped that she had left physic and physicians behind, and would spend the remainder of her brief life undisturbed by nauseating doses; but her kind friends, unwilling to see her suffer and sink without medical attention, at length prevailed on her to see and consult with me. After listening to her story, which was related with clearness, precision and an earnestness of feeling, which deeply enlisted my sympathies, I perceived that it was necessary to make a strong impression on her mind, in order that her prejudices and fears might be thrown aside, and especially if I hoped to accomplish any good in treating the case. Accordingly, I entered into a thorough interrogation in relation to the history, treatment and the present condition, and then gave the most positive assurance that there was no fatal malady preying on her system; that she needed no nauseating doses, no reducing medicines, but on the contrary, she required a plain, substantial, nourishing diet, some gentle and pleasant tonics; she needed to look out from under the cloud which had hung around her for so many weary months; her appetite should be tempted with some good ham or tender beefsteak, a partridge or squirrel nicely broiled, a well baked or roasted potatoe, some well baked light bread or light rolls with a little fresh butter, a soft boiled egg, and other articles of a similar kind. The transition from the apothecary's bottles and boxes and lotions to a well supplied dining table was sudden, and as pleasant as sudden, and as effectual as pleasant. In the course of three or four weeks, I gave her some three or four ounces of sweet spirits of nitre, partly as a diuretic and partly as a placebo. Her appetite, which had been very poor for more than a year, soon returned, her strength increased, her spirits became more buoyant; the health and charms, and hopes and joys of early life gathered once more around her, and she is now living, a happy wife and mother, with the prospect of another addition to the family.

Such cases and such mistakes on the part of physicians are of frequent occurrence.

The physical system, dragged down by frequent childbirth, should have been sustained and braced rather than still farther reduced by active depleting medicines; her spirits depressed by the loss of children, needed the cordial of a rational encouragement; and the effort to produce abortion was a most serious, and might have proved a fatal blunder. Had the physician succeeded in his efforts, her health would doubtless have suffered far more evil consequences than resulted from bearing the child to its full period.

Such facts should teach the utmost caution in pronouncing on the inability of women, however delicate, to live through gestation and give birth to a living child. In the vast majority of cases, the effects resulting from abortions are far more serious and destructive to health than the regular growth and birth of a living child.

